# Effect of under triage on early mortality after major pediatric trauma: a registry-based propensity score matching analysis

**DOI:** 10.1186/s13017-020-00345-w

**Published:** 2021-01-07

**Authors:** François-Xavier Ageron, Jordan Porteaud, Jean-Noël Evain, Anne Millet, Jules Greze, Cécile Vallot, Albrice Levrat, Guillaume Mortamet, Pierre Bouzat, For the TRENAU Group

**Affiliations:** 1 RENAU Northern French Alps Emergency Network, Public Health Department, Annecy Hospital, F-74000 Annecy, France; 2 Grenoble Alps Trauma Center, Department of Anesthesiology and Intensive Care Medicine, Grenoble University Hospital, F-38000 Grenoble, France; 3 Department of Pediatric Care, Pediatric Intensive Care Unit, Grenoble University Hospital, F-38000 Grenoble, France; 4 Department of Intensive Care, Annecy Hospital, F-74000 Annecy, France; 5 Grenoble Alps University, F-38000 Grenoble, France; 6 Grenoble Alpes Trauma Centre, Pôle d’Anesthésie-Réanimation, Hôpital Albert Michallon, BP 217, F-38043 Grenoble, France

**Keywords:** Major pediatric trauma, Under triage, Mortality, Trauma system, Propensity score

## Abstract

**Background:**

Little is known about the effect of under triage on early mortality in trauma in a pediatric population. Our objective is to describe the effect of under triage on 24-h mortality after major pediatric trauma in a regional trauma system.

**Methods:**

This cohort study was conducted from January 2009 to December 2017. Data were obtained from the registry of the Northern French Alps Trauma System. The network guidelines triage pediatric trauma patients according to an algorithm shared with adult patients. Under triage was defined by the number of pediatric trauma patients that required specialized trauma care transported to a non-level I pediatric trauma center on the total number of injured patients with critical resource use. The effect of under triage on 24-h mortality was assessed with inverse probability treatment weighting (IPTW) and a propensity score (Ps) matching analysis.

**Results:**

A total of 1143 pediatric patients were included (mean [SD], age 10 [5] years), mainly after a blunt trauma (1130 [99%]). Of the children, 402 (35%) had an ISS higher than 15 and 547 (48%) required specialized trauma care. Nineteen (1.7%) patients died within 24 h. Under triage rate was 33% based on the need of specialized trauma care. Under triage of children requiring specialized trauma care increased the risk of death in IPTW (risk difference 6.0 [95% CI 1.3–10.7]) and Ps matching analyses (risk difference 3.1 [95% CI 0.8–5.4]).

**Conclusions:**

In a regional inclusive trauma system, under triage increased the risk of early death after pediatric major trauma.

## Background

The implementation of trauma systems has been advocated worldwide to reduce mortality after severe trauma [1]. These systems rely on designated trauma centers and standardized field triage to provide appropriate care according to the patient’s needs [2]. Field triage protocols aim for the lowest under triage possible, i.e., the admission of a severely injured patient to a non-specialized trauma center [3]. These principles apply all the same to children [4]. Indeed, a beneficial effect on mortality was demonstrated when children and adolescents were treated in pediatric trauma centers [5, 6]. These concepts plead for a prompt and accurate initial clinical assessment and triage of pediatric patients to limit preventable deaths in children with severe trauma [7].

The available evidence on pediatric triage is scarce. Most triage protocols were adapted from adult algorithms in countries where prehospital care is performed by paramedics [8]. However, the anatomic and physiologic characteristics of children makes prehospital triage a challenge [9]. A recent literature review documented the use of specific field triage protocols to children carried a high risk of under triage [10]. However, robust data on the effect of under triage on mortality after major trauma are still lacking. The Northern French Alps trauma system (TRENAU) is an inclusive regional trauma system implemented in the French Alps with a physician-staffed field triage procedure combining a grading system with an algorithm for triage [11]. Pediatric adaptations of the grading system are performed by the on-scene physician for several items; however, the triage of grade A, B, or C patients is performed the same way as adults. We thus hypothesized that the current TRENAU triage algorithm could generate a high under triage rate in pediatric patients with a potential deleterious effect on mortality. The main objective of the study was to assess the effect of under triage on early mortality in a pediatric trauma population.

## Methods

We performed an observational study following the Strengthening the Reporting of Observational Studies in Epidemiology (STROBE) guidelines. A checklist for this cohort study is provided in eTable 1 in the Supplement.

### Patients and data collection

Between January 2009 and December 2017, consecutive pediatric patients (≤ 15-year-old) included in the TRENAU registry for a suspicion of severe trauma were analyzed. The TRENAU registry has obtained approval from the institutional review board (Comité de Protection des Personnes, Clermont-Ferrand), the Advisory Committee for Information Processing in Health Research (Comité Consultatif Pour le Traitement de l’Information en Matière de Recherche Dans le Domaine de la Santé, 15.038bis), and from the National Data Protection Agency (Commission Nationale de l’Informatique et des Libertés 915372), waiving the need for informed consent. We excluded pediatric patients with on-scene death and pediatric patients initially managed outside the TRENAU network. Regarding epidemiological, clinical, physiological, and biological variables, the TRENAU registry followed the revised version of the Utstein template for uniform reporting of data following major trauma [12]. Calculation of the 2005 version of injury severity score (ISS) was also performed [13].

### Setting

All pediatric patients were admitted in one of the fourteen hospitals of the TRENAU network that covered a regional area in the French Alps of 2 million inhabitants with high seasonal variation (8 million tourists each year). Only one hospital is a level I pediatric trauma center. Other hospitals are non-pediatric trauma centers. One hospital is a level I adult trauma center with a pediatric standard ward and one hospital is a level II adult trauma center with a pediatric standard ward. The remaining eleven hospitals are level III trauma centers with no pediatric facility. If a major trauma is suspected, a specialized physician correspondent in the regional EMS call center dispatches a physician-staffed ambulance. Otherwise, a paramedic staffed ambulance of the fire department handles the case. On scene, patients are graded from categories A to C (supplemental efigure 1) according to physiological, anatomical, and anamnestic criteria by a prehospital emergency physician. The algorithm takes into account the specifics of pediatric vital signs (heart rate and arterial blood pressure according to age), the level of consciousness (pediatric Glasgow coma score) and the height of fall (twice the height of the child). According to this protocol, an algorithm is applied to dispatch pediatric patients to dedicated trauma centers in the same way as adult patients (supplemental eFigure 2). The protocol is not applied when the medical team is not available, when a physician does not comply with the procedure or when initial assessment by the fire department ambulance does not indicate a high level of acuity.

### Outcomes and triage definitions

The primary outcome was death of any cause at 24 h, based on the hypothesis that increasing under triage is associated with a higher 24-h mortality. Early death was chosen as a primary outcome to assess the effect of under triage since under triage might affect the use of timely early critical resources that were immediately available in level I pediatric trauma center. Injured children have also higher incidence of early mortality compared to adults [14].

Secondary outcomes were in-hospital death, length of stay in intensive care unit (ICU), and length of stay in hospital. Major trauma patients are usually defined by an ISS > 15 [15]. Since ISS predicts mainly mortality [13], several studies rely on the need of early critical resource use to assess triage accuracy [10]. In our study, trauma severity was defined by the need of specialized trauma care such as pediatric ICU admission, non-orthopedic surgery, embolization or transfusion within 24 h. Under triage was defined by the number of injured patients with the need of specialized trauma care transported to a non-level I pediatric trauma center on the total number of injured patients that needed specialized trauma care. Over triage was defined by the number of patients without critical resource use initially transported to a level I pediatric trauma center with trauma team activation on the total number of patients without critical resource use. We also assessed under triage and over triage using the ISS definition as a sensitivity analysis.

### Statistics

Continuous variables were expressed as mean and standard deviation (SD) or as median and interquartile range (IQR). Categorical variables were expressed as frequency and percentage with 95% confidence interval (95% CI). First, the accuracy of the field triage was estimated by sensitivity, specificity, positive predictive value, and negative predictive value. Sensitivity corresponds to the probability of the need for specialized trauma care and/or major injury (ISS > 15) when transported to level I pediatric trauma center. Specificity is the probability of no need for a specialized trauma care or not being severely injured and not transported to a level I pediatric trauma center. Positive predictive value is the probability of being transported to a level I pediatric trauma center when patient needs critical care resources or is severely injured. Negative predictive value is the probability of not being transported to a level I pediatric trauma center when patient does not need specialized trauma care or is not severely injured. Under triage corresponds to 1-sensitivity. Over triage corresponds to 1-specificity, except for the patient to be transported to a level I center without trauma team activation and admitted to general emergency ward.

Second, the impact of under triage on the primary outcome (death at 24 h) and its impact on secondary outcomes were assessed. Crude analysis compared outcomes in under triaged patients to patients correctly triaged among patients needing specialized trauma care. To reduce biased estimate, inverse probability treatment weighting (IPTW) method and propensity score matching analysis were performed. Both methods are based on propensity score (Ps) accounting for known confounders. In non-randomized trials, confounder is likely to influence exposure allocation of being under triaged. The propensity score estimated the probability of being under triaged given known confounders by using the regression equation of a logistic model. The analysis included confounders associated to the outcome and the exposure in a parsimonious way by selecting only variables at baseline that cannot be on the causal pathway. Selected confounders were age, first prehospital vital signs (systolic blood pressure, heart rate, Glasgow coma scale), and severity of injury with Injury Severity Score (ISS). IPTW corresponds to weighting the outcome measured by the inverse of the probability to be assigned in the exposure allocation of being under triaged, i.e., the propensity score. In practice, outcome is weighted by 1/Ps for under triaged patients and 1/(1-Ps) for non-under triaged patients. Propensity score matching analysis matches each under triaged patient with non-under triaged patient based on the nearest neighbor propensity score with a caliper of 0.1. Both methods allow to estimate the average treatment effect which corresponding to the risk difference between outcome of under triaged patient and non-under triaged patient. We plotted the risk difference for each outcome and each method used. IPTW and Ps match were used as sensitivity analysis to one another. The registry-based study design predetermines the sample size. Post hoc power calculation was based on risk difference observed in the propensity score matching analysis using Pearson’s chi-squared test. All analyses were performed using STATA software (version 14.0; Stata Corp., College Station, TX, USA).

### Missing data

There was no loss to follow for the outcome and between 0 and 37% missing values for predictors used in the propensity score. A multiple imputation by chained equations to fill in missing value of predictors was performed. Twenty imputed datasets were generated and 1064 missing values (19%) for 466 incomplete observations were imputed.

## Results

Between 2009 and 2017, 1180 consecutive pediatric patients were admitted in the TRENAU network with a suspicion of severe trauma. Four children died on scene and 33 were managed by a prehospital team outside the TRENAU network (flow chart in supplemental eFigure 3). Table 1 shows the demographic characteristics of the 1143 analyzed patients. Among these, 547 (48%) patients required specialized trauma care and 402 (35%) patients had an injury pattern with ISS > 15 (Table 2). Nineteen (1.7%) patients died within 24 h and 25 (2.2%) patients died in-hospital. Among patients who died within hospital, three patients were considered to have preventable death by FXA and PB (one patient had a severe TBI and two patients died from acute hemorrhage). The graded triage protocol was only applied to 632 (55%) patients. Clinicians graded 50 patients (4%) into grade A, 159 patients (14%) into grade B, and 423 patients (37%) into grade C. Analysis revealed the following reasons to not apply the triage tool: absence of the medical team/spontaneous presentation to hospital (188 patients, 17%) and omission by the physician on scene (323 patients, 28%). Table 3 shows the comparison between graded patients and non-graded patients in terms of demographic and physiologic characteristics. Non-graded patients were more likely to have mountain accidents and intermediate traumatic brain injury. Inter-hospital transfers occurred more frequently for non-graded patients (152 patients [24%] in the non-graded group versus 46 patients [9%] in the graded group; *P* < 0.001) and time from admission to transfer tended to be longer (non-graded patients = 447 min versus graded patients = 236 min; *P* = 0.545).

**Table 1:** Characteristics of trauma children.

Characteristics	*N* = 1143	Missing value, N (%)
Age, years (mean, SD)	10 ± 5	0
Age, N (%)		
0–2	75 (7)	
2–6	175 (15)	
6–10	216 (19)	
10–15	680 (59)	
Sex male, N (%)	745 (65)	0
Mechanism of injury, N (%)		10 (1)
Road traffic accident	401 (35)	
Falls	582 (51)	
Stabbing, gunshot	13 (1)	
Hit by object or person	120 (11)	
Mountain accidents	449 (39)	
Skiing accidents	335 (29)	
Helicopter transport, N (%)	467 (41)	45 (5)
Prehospital GCS, N (%)		252 (22)
15–14	688 (77)	
13–9	107 (12)	
8–3	99 (11)	
Prehospital SBP, mean (SD)	116 (21)	421 (37)
Prehospital SBP < 70 + 2 × age, N (%)	25 (2)	
Prehospital oxygen saturation < 90%, N (%)	27 (2)	477 (42)
ISS, mean (SD)	12 (10)	16 (1)
ISS, N (%)		
< 16	733 (65)	
16–24	229 (20)	
25–34	128 (11)	
> 34	40 (4)	
Overall AIS score ≥ 3, number (%)	644 (56)	16 (1)
Head AIS ≥ 3	295 (26)	
Chest AIS ≥ 3	212 (19)	
Abdomen AIS ≥ 3	144 (13)	
Limbs AIS ≥ 3	184 (16)	
Multiple AIS score ≥ 3, number (%)		
2	130 (11)	
≥ 3	33 (3)	

*GCS* Glasgow coma scale, *SBP* systolic blood pressure, *ISS* Injury Severity Score, *AIS* Abbreviated Injury Scale

**Table 2:** Triage characteristics and outcomes

	*N*	% (95% CI)
Pediatric trauma center level I admission	657	57 (55–60)
ISS > 15	402	35 (32–38)
Need for specialized trauma care*	547	48 (45–51)
24-h death	19	1.7 (1.1–2.6)
In-hospital death	25	2.2 (1.5–3.2)
ICU length of stay, median [IQR]	3 [2–6]	
Hospital length of stay, median [IQR]	4 [2–9]	
Causes of death		
Traumatic brain injury	14	56 (36–75)
Hemorrhage	4	16 (6–37)
Multi-organ failure	2	8 (2–28)
Anoxia	1	4 (1–25)
Unknown	4	16 (6–37)

*ISS* Injury Severity Score, *OCU* intensive care unit

*Critical resource use within 24 h (pediatric ICU admission, non-orthopedic surgery, transfusion, or embolization)

**Table 3:** Univariate analysis according to whether children were graded using an on-scene triage procedure (graded group; *n* = 632 patients) or not (non-graded group; *n* = 511 patients)

Variable	Graded group *N* = 632 patients	Non-graded group *N* = 511 patients	*P* value
Mean age, years (SD)	10 ± 5	10 ± 5	0.084
Sex male, N (%)	421 (67)	324 (63)	0.206
Mechanism of injury, N (%)			
Road traffic accidents	255 (40)	146 (29)	< 0.001
Falls	127 (20)	115 (23)	0.383
Skiing accidents	172 (27)	163 (32)	0.103
Other mountain accidents	59 (10)	55 (11)	0.488
Other	8 (1)	17 (3)	0.024
Initial GCS, N (%)			
15 to 14	492 (78)	379 (74)	0.045
13 to 9	80 (13)	92 (18)	
8 to 3	59 (9)	41 (8)	
Initial SBP < 70 + 2 × age(years), N (%)	13 (2)	9 (2)	0.707
Initial assessment of SpO_2_ < 90%, N (%)	15 (2)	12 (2)	0.905
ISS ≥ 16, N (%)	211 (33)	191 (37)	0.183
Need for specialized trauma care ^a^, N (%)	302 (48)	245 (48)	0.968
Death at 24 h, N (%)	15 (2)	4 (1)	0.035
Death at day 28, N (%)	19 (3)	6 (1)	0.034

*GCS* Glasgow coma scale, *SBP* systolic blood pressure, *ISS* Injury Severity Score

^a^ Critical resource use within 24 h (pediatric ICU admission, non-orthopedic surgery, transfusion, or embolization)

Table 4 presents respectively the under triage and over triage rates based on either to the ISS-threshold > 15 or the resource-based definition of trauma severity. Sensitivities of the field triage protocol to identify the need for specialized trauma care or severely injured (ISS > 15) children were 67% and 57% respectively. As a result, under triage rates were 33% based on the need of specialized trauma care and 43% based on the ISS definition of severity (ISS > 15).

**Table 4:** Accuracy of field triage according to the need of critical resources or severe injury with ISS > 15

	Sensibility % (95% CI)	Specificity % (95% CI)	Under triage % (95% CI)	Over triage % (95% CI)	PPV % (95% CI)	NPV % (95% CI)
Need of specialized trauma care*	67 (63–71)	51 (47–56)	33 (29–37)	36 (32–40)	58 (52–60)	63 (59–67)
ISS > 15	57 (52–62)	43 (39–46)	43 (38–48)	44 (40–47)	35 (32–39)	65 (60–69)

*Critical resource use within 24 h (pediatric ICU admission, non-orthopedic surgery, transfusion, or embolization)

**Fig. 1:**
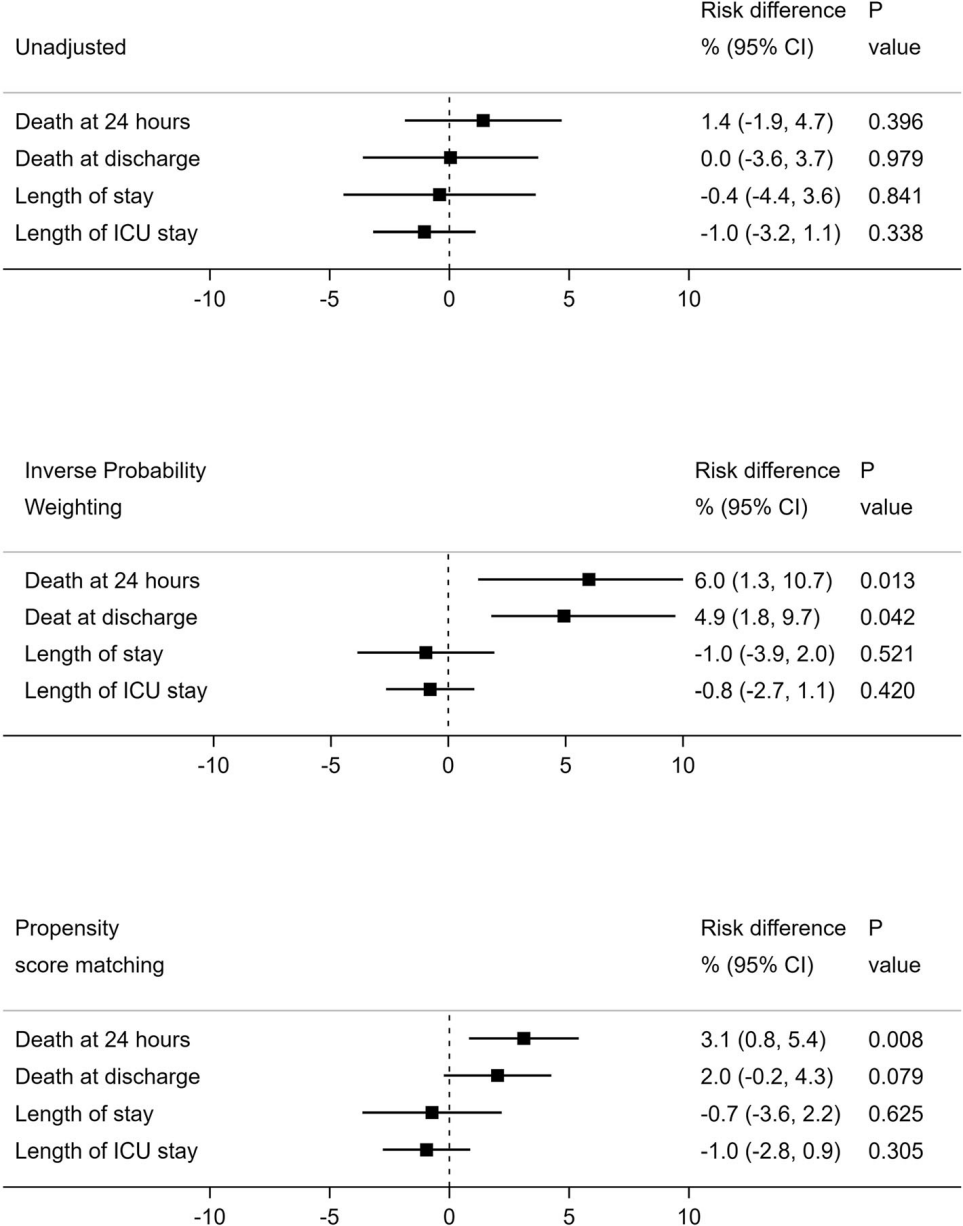
Crude, inverse probability treatment weighting (IPTW) method, and propensity score (Ps) matching analyses to assess the risk of early death in under triaged children according to the need of specialized trauma care

**Fig. 2:**
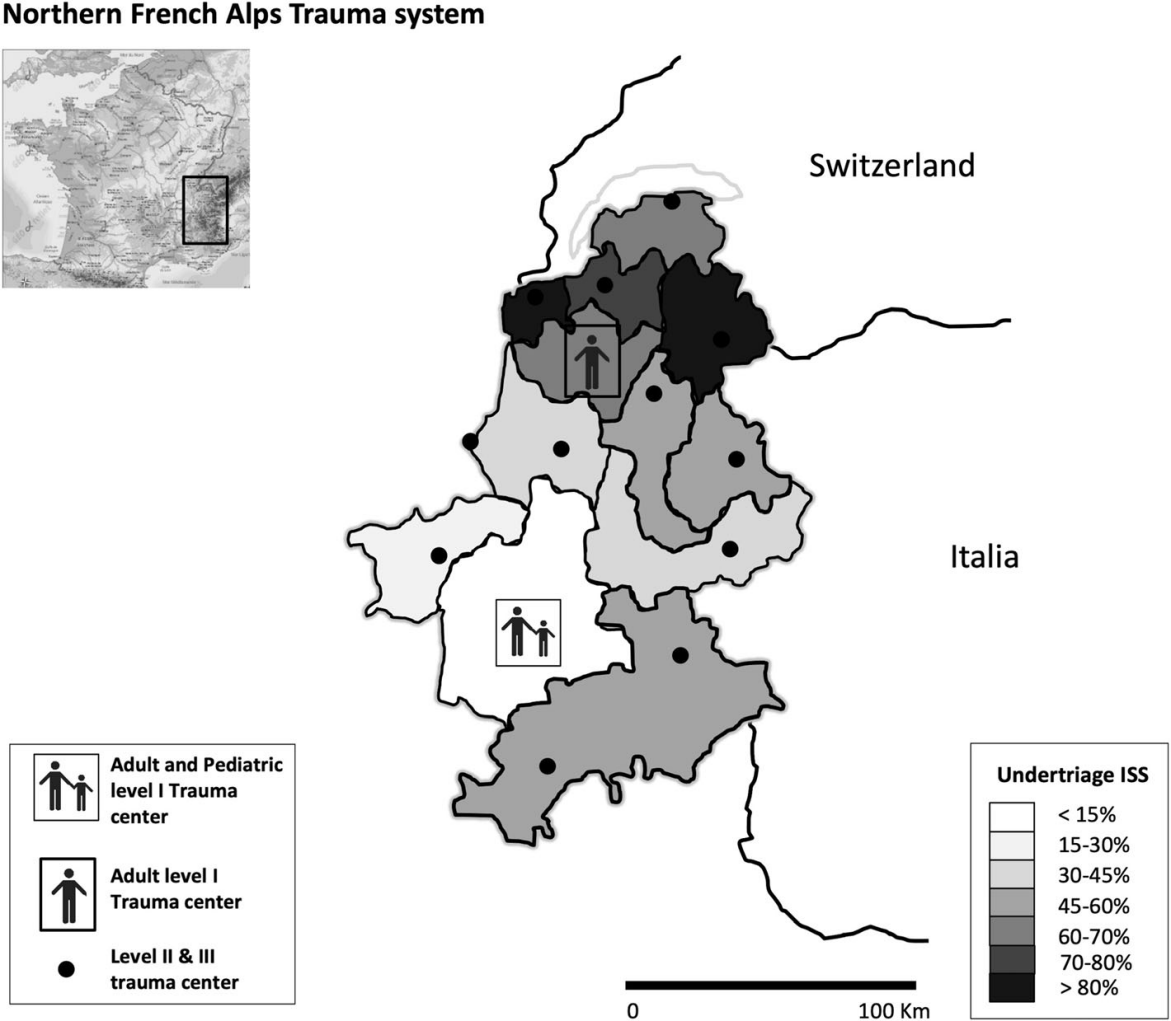
Repartition of under triage rates across the TRENAU administrative sub-territories

Based on the resource-use definition, the occurrence of under triage of children with a proven need for specialized trauma care increased the risk of early death in IPTW and Ps matching analysis (risk difference 6.0 [95% CI 1.3–10.7] and 3.1 [95% CI 0.8–5.4], respectively, see Fig. 1). In this case, the under triage was associated with a higher risk of in-hospital death (IPTW: risk difference 4.9 [95% CI 1.8–9.7]). Based on the resource use definition, hospital and ICU length of stay were similar between under triaged and non-under triaged patients. Based on the ISS > 15 threshold, the occurrence of under triage only increased the risk of early death in IPTW analysis (risk difference 9.3 [95% CI 0.0–18.6]). Ps matching showed a non-statistical increase in the risk of early death equal to 3% [95% CI − 2.1–8.1] (supplemental efigure 4). The post hoc power calculation using risk difference observed in the propensity score matching analysis demonstrated a power of 68% (risk alpha 5%). Figure 2 provides a possible explanation for the observed under triage in this pediatric trauma cohort. According to Fig. 2, in the TRENAU network, the risk of under triage increased with increasing distance of the accident to the level I pediatric trauma center.

## Discussion

In the TRENAU regional trauma system, applying a shared algorithm for the triage of major trauma in children and adults alike led to a high under triage rate in children. Under triage was 33% based on a resource-use and 43% based on an ISS > 15 threshold. Inverse probability weighting and propensity score matching analyses demonstrated an association between under triage and increased 24 h all cause in-hospital mortality.

Since the publication by the American College of Surgeon–Committee on Trauma (ACS-COT) center categorization in 1976, the trauma community uses under and over triage rates as surrogate markers to assess trauma system efficiency [16]. Any trauma system attempts to keep under triage as low as possible while avoiding over triage [1]. The recommended target for under triage is 5% according to the ACS-COT guidelines. The present study demonstrated a very high under triage rates based on resource or ISS definitions respectively. In a recent review including five studies with a combined number of 1222 pediatric patients requiring specialized trauma care, the sensitivity of the prehospital triage tools ranged from 49.1 to 87.3% to correctly identify pediatric major trauma patients. This corresponded to under triage rates between 12.7 and 50.9% [10]. An under triage higher than 20% was also reported in the Western USA between 2006 and 2008 for children 0 to 10 years of age and 11 to 20 years of age with a major trauma (ISS > 15) [17]. Similarly, 21.7% of pediatric major trauma patients (ISS > 15) were under triaged using the 2009–2013 nationwide emergency department sample in the USA [18]. It seems that high under triage rates are common in the pediatric population in diverse settings [19]. In fact, several studies reported unacceptable under triage rates even when using specific pediatric algorithms [10]. These observations seem to mirror the complexities of the triage process itself between the constraints of the triage tool, prehospital provider experience and judgement, and trauma center proximity and capacity [20]. In our study, 16% of trauma children were not transported by the prehospital emergency medical service (EMS) and, even when a physician-staffed ambulance was dispatched, 29% of our pediatric population did not benefit from the triage protocol. The geographical analysis confirmed that increasing distance from the scene to the level I pediatric trauma center carries the risk of under triage.

The effect of under triage on mortality is well established for adult trauma cohorts. Historic studies in North America documented an increased mortality when major trauma patients were not directly transferred to the appropriate trauma center [21, 22]. For instance, using the Glue Grant Trauma Database of severely injured patients, the odds of death were 3.8 times greater (95% CI 1.6–9.0) when patients were initially triaged to a non-specialized facility [21]. Data on pediatric populations are limited. In the largest retrospective study in the USA, a beneficial effect on mortality was observed when children were treated in pediatric trauma centers but, in stratified analyses, this benefit was only observed in younger children (5 years and younger, odds ratio, 1.78; 95% CI 1.05–3.40) [6]. In Europe, the German trauma system also advocates a multidisciplinary approach including pediatric physicians, trauma surgeons, and pediatric intensive care physicians for pediatric trauma management [23]. However, to our knowledge, no specific study was done to explore the association between under triage and early mortality in the German trauma system. The present study attempted to obtain better control of potential confounders with the use of inversed probability treatment weighting and propensity score matching analyses. This approach may provide a more robust assessment of the effect of under triage on early mortality and strongly suggests a reduction in mortality if children are appropriately triaged to pediatric level I trauma centers.

One avenue to explain the observed high under triage rate in this study lies in the specifics of the TRENAU triage tool. With regard to the use in children, the tool integrates adaption of physiological and amnestic criteria. However, in a recent retrospective study of trauma triage in children in the USA, the physiologic criteria showed only a moderate predictive capacity to appropriately determine a trauma center need for children [24]. Specific pediatric trauma scores facilitate the decision process for pediatric trauma patients. For example, the pediatric trauma score was developed to predict injury severity and mortality after pediatric major trauma [25]. However, it is complex to calculate on scene and does not show any advantage in comparison to the revised trauma score [26]. The pediatric trauma triage checklist was supposed to make the pediatric trauma score more “user friendly” and in consequence implemented in many trauma centers in the USA [27]. Unfortunately, a recent systematic review could not demonstrate a reduction in under triage based on this tool [10]. Triage of children after major trauma remains challenging, and a high proportion of injured children is currently misclassified by existing triage protocols resulting in potential deleterious effect on mortality. Based on the results of this work, the TRENAU tool was modified to improve triage of children. The algorithm now recommends a systematic admission to a pediatric level I center for grades A and B patients and all patients under the age of 3 years. Whether these changes will be result in lower under triage in the TRENAU network remains to be studied.

The authors acknowledge several limits of the present work. First, random measurement errors of blood pressure, heart rate, Glasgow coma scale, or ISS could lead to a bias toward the null for the regression coefficient used to estimate the Ps. Since the Ps range could be reduced, it might affect the matching process. The use of IPTW without matching to complete the analysis limited this bias since both methods showed similar results. On the other hand, IPTW could have been biased by extreme weight and increase risk difference. Since Ps matching is less impacted by extreme weight, both methods were used as sensitivity analyses for one another [28]. Second, the authors used Ps to balance known and unknown confounders, but cannot exclude that potential unknown confounders affect the results. The objective of the study was to assess the harmfulness of under triage to justify corrective measures. The objective was not to obtain a precise measure of effect. Third, the authors did not report loss to follow up for outcome, but missing values for predictors of the Ps, but performed multiple imputation with the assumption that missing values occurred at random. Even if multiple imputation bias tends to be smaller that complete case analysis, bias away from the null might affect propensity score estimation. Fourth, the number of death and the total sample size was relatively small, which limited the statistical power of the study (68%) and limited the interpretation of non-statistically significant results. As a result, sensitivity analysis based on the ISS criterion included only 402 patients was under powered to detect a 3% difference in mortality. Unfortunately, this limitation is common in pediatric trauma cohorts considering their overall low mortality [14]. Finally, our results concern data in a specific mountainous area and cannot be apply in an urban trauma system.

## Conclusions

In a regional inclusive trauma system, a shared universal trauma triage algorithm for adults and children alike with pediatric specifications was associated with a higher proportion of under triaged pediatric patients. A propensity score matching analysis demonstrated that under triage increased 24-h mortality. These findings plead for the implementation of specific pediatric trauma algorithm for on scene triage to reduce early mortality.

## Supplementary Information

**Additional file 1: eTable 1**: Checklist of the Strengthening the Reporting of Observational Studies in Epidemiology (STROBE) guidelines. **eFigure1**: Grading protocol of severe trauma children in the TRENAU network. **eFigure 2**: Algorithm for the direction of severe trauma patients (adluts and children) in the TRENAU network. **eFigure 3**: Flowchart of the study. **eFigure 4**: Crude, inverse probability treatment weighting (IPTW) method and propensity score (Ps) matching analyses to assess the risk of early death in under triaged children according to the Injury Severity Score definition.

## Data Availability

The datasets generated and/or analyzed during the current study are available from the corresponding author on reasonable request.
